# Comparative study of efficiency and characteristics of FMT and DRT installed in human cadavers for round-window stimulation

**DOI:** 10.1038/s41598-021-95456-y

**Published:** 2021-08-18

**Authors:** Dong Ho Shin, Jong Hoon Kim, Peter Gottlieb, Yona Vaisbuch, Sunil Puria, Jin-Ho Cho, Ki Woong Seong

**Affiliations:** 1 Institute of Biomedical Engineering Research, Kyungpook National University, Daegu, 41944 Korea; 2 Department of Mechanical Engineering, Stanford University, Stanford, CA 94305 USA; 3 Otolaryngology Head and Neck Department, Rambam Medical Center, 31096 Haifa, Israel; 4 Department of Biomedical Engineering, Kyungpook National University Hospital, Daegu, 41944 Korea; 5 Present Address: Eaton Peabody Laboratory, Department of Otolaryngology-Head and Neck Surgery, Harvard Medical School, Massachusetts Eye and Ear Infirmary, Boston, MA 02114 USA

**Keywords:** Biomedical engineering, Rehabilitation

## Abstract

Acoustic hearing aids generate amplified sound in the ear canal, and they are the standard of care for patients with mild to moderate sensorineural hearing loss. However, because of their limited frequency bandwidth, gain, and feedback, there is substantial room for improvement. Active middle ear implants, which directly vibrate the middle ear and cochlea, are an alternative approach to conventional acoustic hearing aids. They provide an opportunity to improve sound quality and speech understanding with amplification rehabilitation. For floating-mass type and direct-rod type (DRT) middle ear transducers, a differential floating-mass transducer (DFMT) and a tri-coil bellows transducer (TCBT), respectively, were fabricated to measure the output characteristics in four human temporal bones. Both were fabricated to have similar output forces per unit input and were placed in four human temporal bones to measure their output performances. The TCBT resulted in higher output than did the DFMT throughout the audible frequency range, and the output was more prominent at lower frequency ranges. In this study, we showed that DRT was a more effective method for round window stimulation. Because of its frequency characteristics and vibration efficiency, this implantation method can be utilized as a driving solution for middle ear implants.

## Introduction

Active middle ear implants (AMEIs) are alternatives to conventional hearing aids, which are effective for sensorineural and mixed hearing loss^1–6^. The first approach used by Finnish scientist Alvar Wilska (1911–1987) was to transmit vibrational energy to the inner ear by using fluctuating magnetic fields and iron particles on the tympanic membrane^4^. The first clinical AMEI was reported by Yanagihara in Japan in 1984 and was used to remove part of the ossicle chain to directly drive the stapes footplate^5^. Therefore, this device could only be applied to patients with middle ear malfunction, because patients cannot use a conventional hearing aid if the implant fails. The Vibrant^®^ Soundbridge™ transfers energy by suspending a small vibrating transducer on intact ossicles and does not cause clinically significant changes to residual hearing. Because of these advantages, the Vibrant^®^ Soundbridge™ has been used in many clinical studies. The device was approved for sensorineural/mixed hearing loss in 2000 by the Food and Drug Administration^4^.

AMEIs compensate for hearing loss by applying amplified vibrations on the auditory pathway. The vibration output is generated by using electromagnetic or piezoelectric transducers; in most instances, electromagnetic-type transducers are appropriate because they do not need highly amplified driving voltages, whereas piezoelectric transducers do. The vibration transducers are classified into direct-rod type transducers (DRTs) and floating-mass type transducers (FMTs)^7^. Some studies categorize vibration transducers as incus driving type transducers and FMTs^7^. The FMT™ (MED-EL) and MET™ (Cochlear, Ltd.) are representative of FMTs and DRTs, respectively; many other types of transducers have also been developed.

In recent studies, round-window (RW) vibroplasty with AMEI vibration transducers has been the focus of investigation because RW stimulation can be used to treat patients with middle ear diseases, such as otosclerosis^8–17^. Over the past 10 years, many studies have verified the effectiveness of RW stimulation, but the vibration transducers used for RW stimulation have been the transducers used in conventional AMEIs. The transducers frequently used to study AMEIs were FMT™ (MED-EL, USA) and MET™ (Cochlear Ltd., AUS), which represent FMTs and DRTs, respectively. Previous studies of oval-window and RW stimulation have shown that both types of transducers have sufficient high-frequency output relative to low-frequency output; thus, they are considered suitable for sensorineural hearing loss compensation. In the low-frequency range, the DRT showed better output than did the FMT in both types of stimulation^7^.

An FMT is composed of springs that connect two masses of a field coil and a magnet. When the weight of the field coil is increased (i.e., when the springs are attached to the ossicles and their weights are added to the field coil), the low-frequency output is greatly reduced. The DFMT proposed by Kim et al.^18^ consists of a differential magnet to which are glued two magnets with the same poles; these magnets focus the magnetic flux emitted in a radial direction from the center of the magnet and the three field coils, thus generating the driving force. This modified FMT-type transducer is able to cancel the external environmental magnetic fields while remaining highly efficient. Although some structures inside the transducer have been modified, its characteristic frequency is similar to the characteristic frequency of the FMT-type transducer because it operates in a floating-mass style^19^.

In contrast, because the field coil of a DRT is fixed to the bony structure of the middle ear cavity wall, this transducer increases only the mass of the magnet on the RW membrane after implantation. Therefore, it causes only a small shift of the resonance peak to a lower frequency; however, it does not introduce any loss in the low-frequency gain. In addition, the DRT can be designed to have a stronger vibration power because it has more space in the mastoid than does the FMT in the middle ear cavity. However, the difficulty level of the procedure used to implant each transducer is considerably different. Because the source of the transducer driving force is different, it is premature to argue which type of transducer is more efficient, regardless of whether the transducers are driven with an identical current or voltage.

The TCBT that was recently proposed by Shin et al.^20^ is almost the same size as the FMT™, and its body is fixed to the cavity wall with the field coil. However, when the differential magnet inside the field coil vibrates with the signal current, the end surface of the elastic bellows that is connected to the magnet vibrates to drive the RW membrane. Therefore, this transducer can be classified as a DRT-type transducer, similar to the MET™ body, because its field coil is fixed. The implantation of this transducer is easier than implantation of the conventional DRT because the TCBT is small and can be installed in the RW niche; moreover, it is unnecessary to use a rigid driving rod because a flexible thin lead wire is connected to the vibrating coil. Considering the advantages and clinical significance of the driving method used for the RW, it is important to compare the characteristics and surgical effectiveness of the new DRT and FMT vibrators that have been recently manufactured to drive the RW.

In this study, we investigated the efficiency of the method used to fix a transducer in an RW stimulation using human cadavers. For this purpose, previously studied DFMTs and TCBTs were designed and fabricated to have the same driving force and electrical characteristics.

## Materials and methods

### Preparation of temporal bones

**Figure 1:**
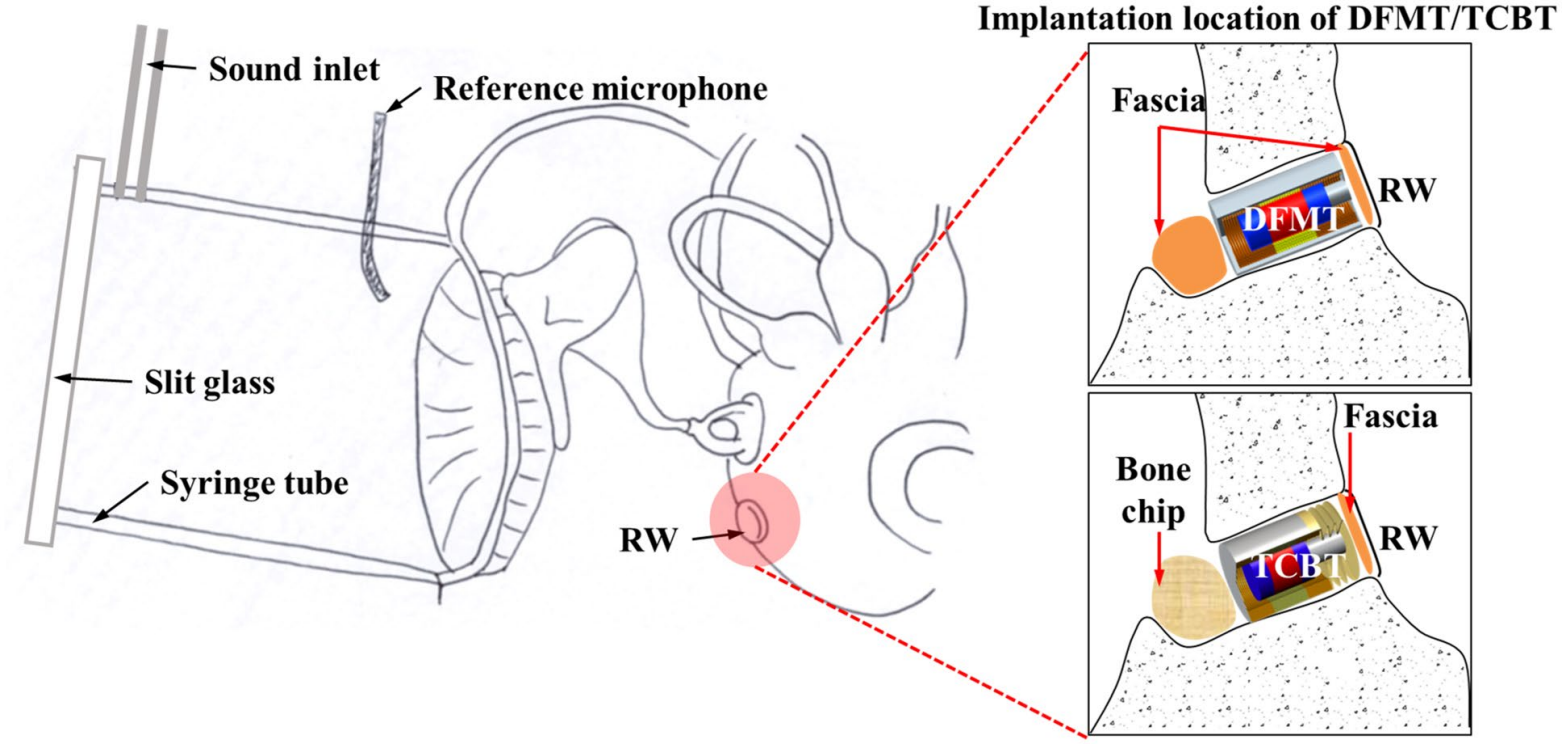
Schematic diagram of temporal bone preparation for cadaver experiments.

To conduct the experiments, four human temporal bones were obtained from the Anatomy Gifts Registry, labeled as TB1, TB2, TB3, and TB4 and stored in a refrigerator. TB1 and TB2 were from a 65-year-old Caucasian man, while TB3 and TB4 were from a 59-year-old Asian man. The temporal bones were dissected to an easy-to-use size for the experiment. A mastoidectomy was performed to secure the angle of the laser delivered to the footplate. Concurrently, drilling was performed to avoid changing the vibration characteristics of the temporal bone due to stapedius muscle damage. The bones near the RW were drilled out to facilitate DFMT and TCBT coupling. RW niche drilling was minimized. An artificial ear canal (AEC) was constructed by attaching a 30-mm-long syringe tube to a 10-mm-diameter tube after complete removal of the ear canal soft tissue, as shown in Fig. 1. At one end of the AEC, a 2-mm-diameter tube was installed for sound input; at the other end of the AEC, a reference microphone was installed to calibrate sound pressure from the sound inlet. The AEC was carefully attached to the tip of a reference microphone and placed approximately 1 mm from the eardrum. The entrance of the AEC was covered with a thin glass slit, and the temporal bone was covered with clay to minimize any changes in characteristics due to evaporative moisture loss. These experiments were conducted at Stanford University (CA, USA). Stanford University generally states that studies involving cadavers or deceased individuals do not meet the definition of human studies^21^. Therefore, the university’s IRB does not require a protocol for temporal bone experiments.

### Vibrational transducers

**Figure 2:**
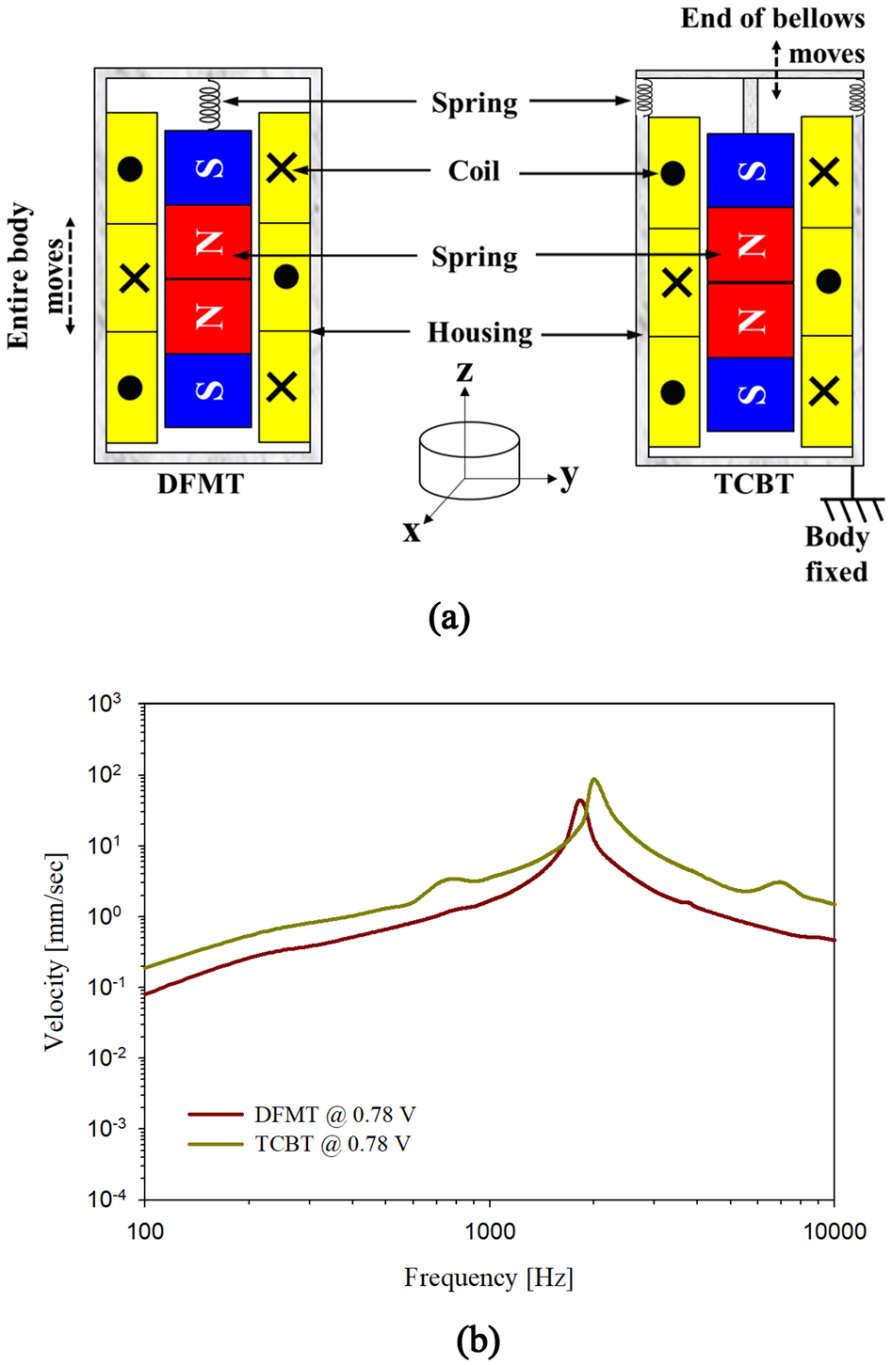
(**a**) Schematic structure showing the composition of the DFMT and TCBT and (**b**) their free-load vibration characteristics.

Two types of vibrational transducers, free-floating and rigidly supported transducers (DFMT and TCBT, respectively), were fabricated. Both were electrodynamic transducers with similar basic operation principles, as shown in previous studies^20,22^. Both transducers consisted of three magnetic poles that were glued at the same pole face; three coils wound around the magnet, thus orienting Lorentz’s force in a consistent direction (Fig. 2a). The total weights of the DFMT and TCBT were identical (25 mg each), and the electrical resistance was 360 Ω in both transducers. Figure 2b shows the vibration characteristics of the transducers driven by 0.78 V. Measurements were performed with the DFMT floating in the air, while the back of the TCBT was fixed to the wall. The resonance frequencies of the DFMT and TCBT were slightly different, 1.8 kHz and 2 kHz, respectively; the vibration amplitude of the TCBT was approximately 6 dB higher than the vibration amplitude of the DFMT.

### Measurement setup

**Figure 3:**
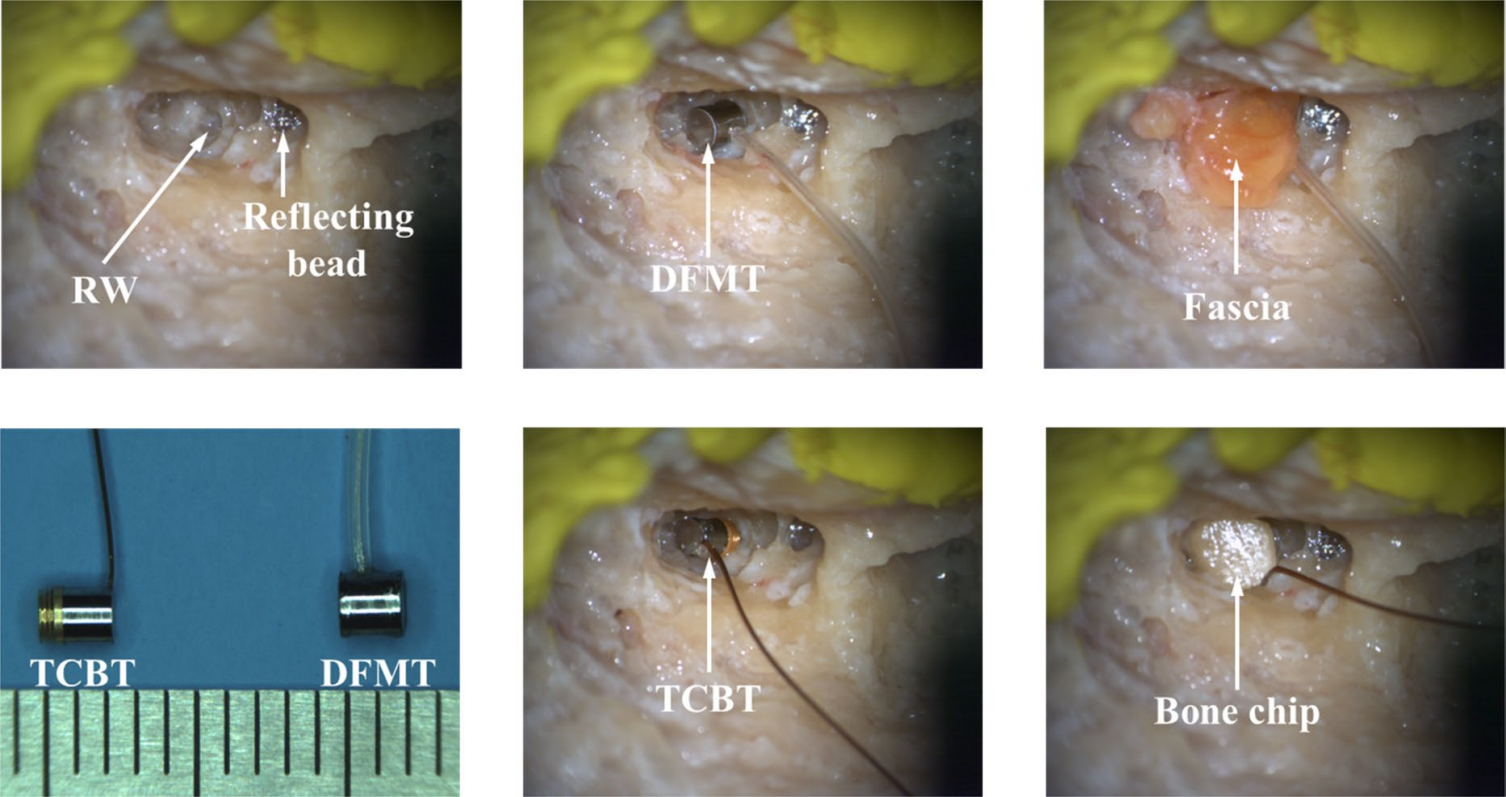
Photographs of the implantation process of DFMT and TCBT.

A measurement automation system (SyncAV-0.26, Stanford University, USA), described in previous studies^20^, was used and the time required for measurement in a cadaver was approximately 30 min. The system had an output for driving a speaker or transducers; it had two inputs for measuring the reference microphone (ER-7C; Etymotic Research Inc., USA) and LDV signal (OFV-551 and OFV-5000; Polytec GmbH, Germany). The temporal bone was fixed, and the vessel was carefully adjusted for the incident angle of the laser and kept constant. A patch of small soft tissue was laid over the RW to prevent punctures to the membrane and to minimize the decrease in vibration transfer efficiency that occurred because of suboptimal transducer implantation. For the DFMT implantation, soft tissue was inserted into the back of the DFMT, thus ensuring that transducers vibrated correctly in the floating state. The DFMT and TCBT implantation processes are shown in Fig. 3. To prevent vibration of the TCBT housing, some bone chips were inserted and supported as rigidly as possible. Before the experiment, the temporal bones were confirmed to have normal vibration characteristics against the sound input, and the loading effects were checked after the implantation of each transducer.

## Results

**Figure 4:**
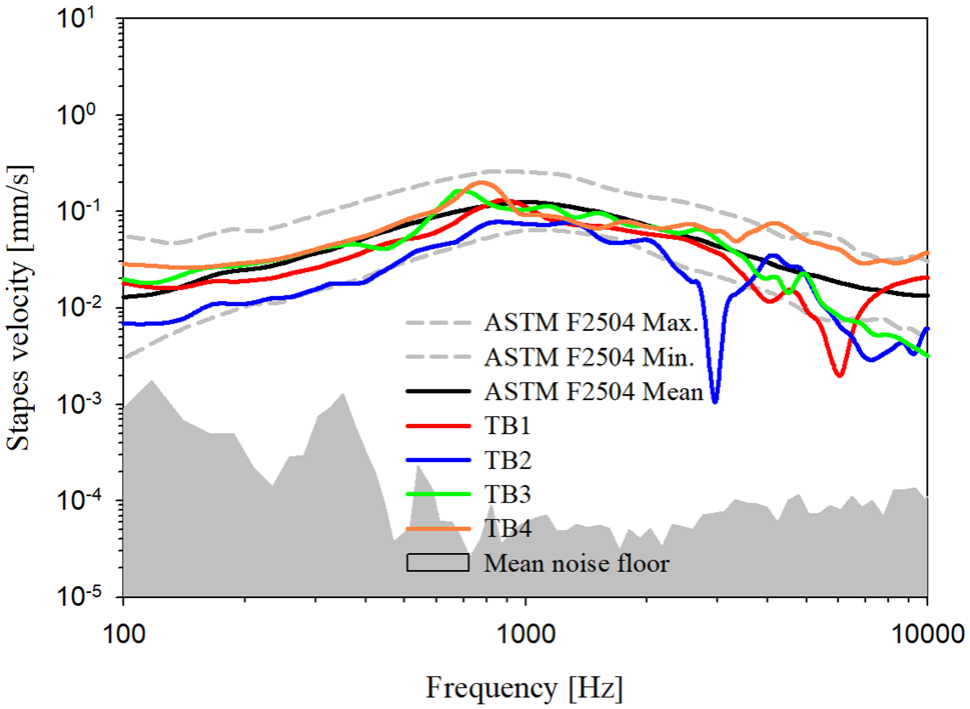
Stapes vibration characteristics of TBs corrected to 94 dB SPL in front of TM.

Figure 4 shows the vibration characteristics of the footplates of the temporal bones for calibrated acoustic stimuli, corrected to 94 dB SPL using the reference microphone installed in front of the eardrum. The vibration characteristics of the temporal bones were similar to the normal vibration characteristics, with reference to ASTM-F2504^23^, and showed a dip at specific frequencies. We assumed that no shortage in testing the differences was present in the output characteristics of the two transducers.

**Figure 5:**
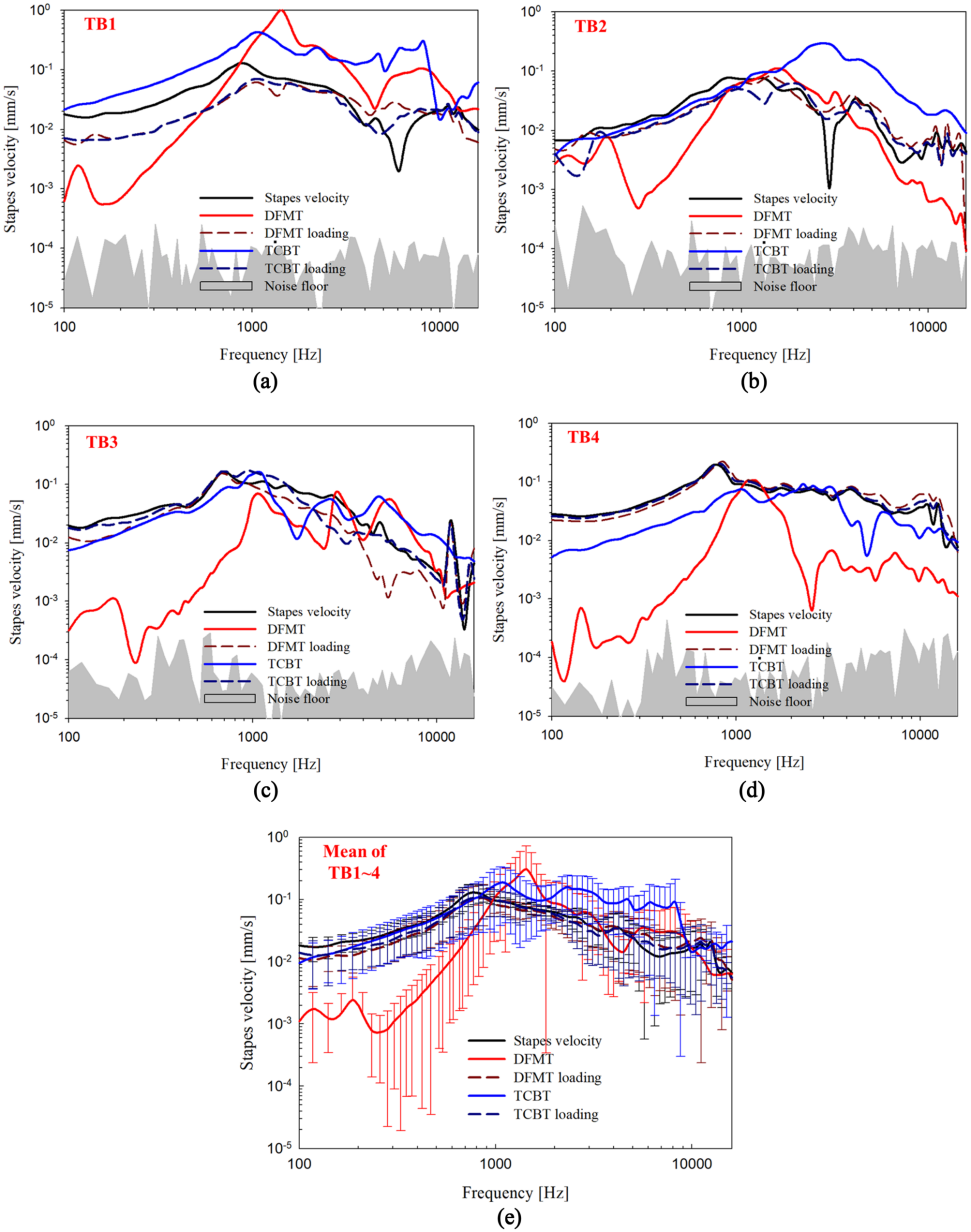
Stapes velocities with 1 Pa of sound pressure before and after the implantation of each transducer and the vibrational output of the transducers driven by 0.78 V. (**a**) TB1, (**b**) TB2, (**c**) TB3, (**d**) TB4, and (**e**) mean of TB1–4; before implantation (black solid line), after DFMT implantation (dark red dashed line), after TCBT implantation (dark blue dashed line), DFMT output (red solid line), and TCBT output (blue solid line).

Figure 5a–d shows the loading effect and output of each transducer driven by 0.78 V; the mean is shown in Fig. 5e. The two transducers were implanted using two different methods, although careful monitoring was performed to ensure that the loading effects were similar; thus, the outputs could be compared when driven with the same energy. Generally, the loading effect of the transducer showed predominant attenuation at high frequencies above 1 kHz because of the transducer masses when two types of transducers were implanted in the ossicles. However, the loading effect of the RW implant showed predominant attenuation at low frequencies, which presumably caused the increased RW stiffness. Overall, the TCBT showed high output characteristics, except at the resonance point of approximately 1 kHz, and the attenuations of the DFMT and TCBT in the low-frequency regions were − 40 dB/octave and − 20 dB/octave, respectively.

The differences in low-frequency output characteristics were as follows: the TCBT housing was wrapped around the field coil and was thus in contact with the bone of the RW niche. The force generated between the inner magnets by the signal current was directly transmitted to the end surface of the bellows and drove the RW membrane. However, the vibrating body housing of the DFMT drove the RW membrane; the movement of the housing was therefore caused by a reaction between the magnet inside the vibrating body and the field coil on the housing side. Because the reaction force was smaller at lower frequencies, the low-frequency gain of the DFMT decreased.

To perform the t-test for Figs. 4 and 5, normality tests were performed using IBM SPSS Statistics 26. The effect size and power calculation according to the number of samples (temporal bones) were calculated using G*Power v3.1.9.7 software. The effect size and power for 4 samples were 0.95 and 0.8, respectively. Figures 4 and 5 show that both Kolmogorov–Smirnov and Shapiro–Wilk results were below the significance level (*p* < 0.05), indicating a lack of normality. Therefore, because number of samples was small and normality was absent, non-parametric tests were performed, including the Mann–Whitney U test (data in Fig. 4 are for two-independent-samples tests) and the Wilcoxon signed-rank test (data in Fig. 5 are for two-related-samples tests). The Mann–Whitney U test was performed between TB1–TB4 data in Fig. 4 and the ASTM F-2504 Mean. The t-test analysis showed that TB1, TB2, and TB4 did not exhibit significant differences because the values of the 2-tailed approximate significance probability in the test statistics were 0.057, 0.557, and 0.051, respectively. Therefore, TB1, TB3, and TB4 were not significantly different from the ASTM F-2504 Mean. However, the value of the approximate 2-tailed significance probability in the test statistic for TB2 was 0.000, indicating a significant difference. Therefore, for TB2, we performed ASTM F-2504 Minimum and Mann–Whitney U test analysis. The t-test findings indicated that TB2 did not show a significant difference (approximate 2-tailed significance probability in the test statistic of 0.638). According to ASTM F-2504, if the vibration characteristic of stapes of temporal bone was distributed between minimum and maximum, the bone is defined as normal temporal bone. Therefore, TB2 also did not significantly differ from ASTM F-2504.

To perform a t-test using the data in Fig. 5, the Wilcoxon signed rank test was performed between DFMT and TCBT for each TB. The t-test result showed a significant difference because the values of the approximate 2-tailed significance probability in the test statistics were all 0.000. There was also a difference in the descriptive statistics.

**Figure 6:**
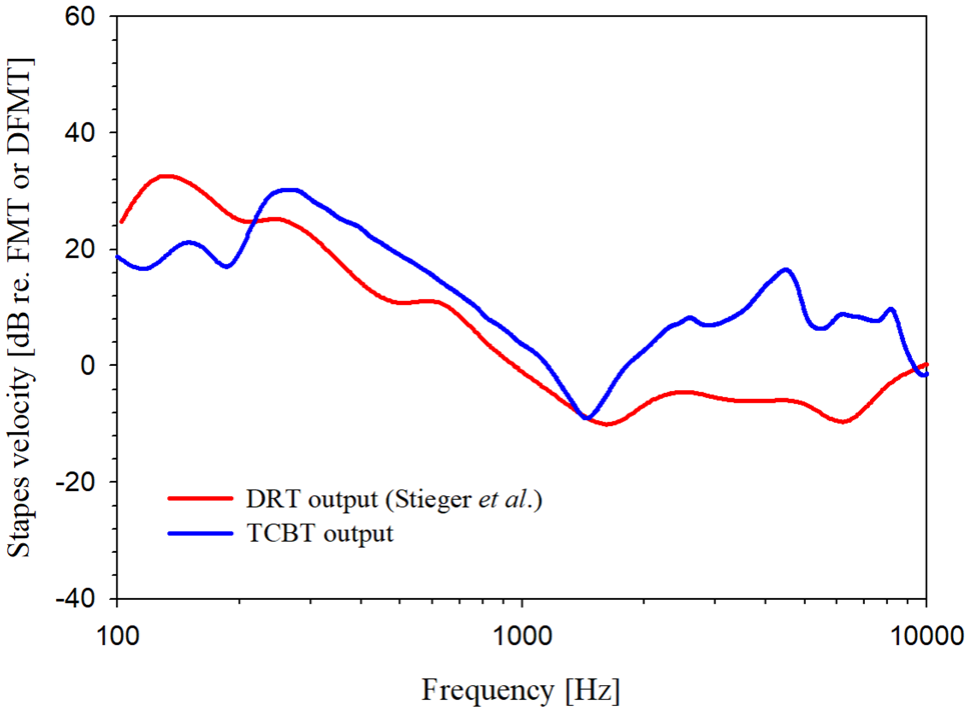
Stapes velocity driven by DRT-type transducer normalized to stapes velocity of FMT-type transducer (red solid: DRT output of oval window driving, blue solid: TCBT output of RW driving).

Figure 6 shows the stapes vibration driven by the DRT normalized to the stapes vibration of the FMT. The red solid line referred to in a previous study of oval window driving^7^ is the output ratio of MET™ to FMT™, and the blue solid line is the output ratio of TCBT to DFMT. The MET™ had a wider indication at low frequencies; this was similar to the experimental results, indicating that the DRT produced superior low-frequency outputs, as determined by comparison with the representative FMT™ of the FMT type transducer. Although the free-load vibration and voltage used in the study^7^ were not described, the output characteristics due to the difference in the driving force transmission methods used for the two transducers can be indirectly compared in our study. For the oval window driving described in the previous study, the stapes velocity due to DRT driving was higher than the stapes velocity of the FMT below 1 kHz, whereas it was 10 dB lower above 1 kHz. In contrast, for the RW driving, the output of the TCBT was superior to the output of the DFMT throughout the measured frequency region, although its resonance was not. In both experiments, the output ratios of the DRT and FMT were similar, indicating that the DRT type of fixation should be used to achieve an efficient low-frequency output.

## Conclusion

In this study, we investigated the type of AMEI transducer that was more efficient based on to the fixation method used. To compare the DRT and FMT types of transducers in RW stimulation, DFMTs and TCBTs (which generate the same electromagnetic force) were fabricated; their outputs were measured using four human temporal bones. The experimental results indicate that the TCBT efficiently delivered the output throughout the frequency range; output was more pronounced at low frequencies. Additionally, the surgical procedure used for TCBT implantation was less complicated than the surgical procedure used for FMT implantation in the RW stimulation, in contrast to the ossicle implantation used for oval-window stimulation. It is thus desirable to use the DRT-type fixation method if the same driving force is used when designing an AMEI output device for RW stimulation. This method should be presumed to efficiently transmit the output.
